# Bioactive molecules from haloarchaea: Scope and prospects for industrial and therapeutic applications

**DOI:** 10.3389/fmicb.2023.1113540

**Published:** 2023-03-31

**Authors:** Jamseel Moopantakath, Madangchanok Imchen, V. T. Anju, Siddhardha Busi, Madhu Dyavaiah, Rosa María Martínez-Espinosa, Ranjith Kumavath

**Affiliations:** ^1^Department of Genomic Science, School of Biological Sciences, Central University of Kerala, Kerala, India; ^2^Department of Microbiology, School of Life Sciences, Pondicherry University, Puducherry, India; ^3^Department of Biochemistry and Molecular Biology, School of Life Sciences, Pondicherry University, Puducherry, India; ^4^Biochemistry, Molecular Biology, Edaphology and Agricultural Chemistry Department, Faculty of Sciences, University of Alicante, Alicante, Spain; ^5^Multidisciplinary Institute for Environmental Studies “Ramón Margalef”, University of Alicante, Alicante, Spain; ^6^Department of Biotechnology, School of Life Sciences, Pondicherry University, Puducherry, India

**Keywords:** haloarchaea, nanoparticles, antimicrobial compound, anticancer, antioxidants, carotenoids, halocins

## Abstract

Marine environments and salty inland ecosystems encompass various environmental conditions, such as extremes of temperature, salinity, pH, pressure, altitude, dry conditions, and nutrient scarcity. The extremely halophilic archaea (also called haloarchaea) are a group of microorganisms requiring high salt concentrations (2–6 M NaCl) for optimal growth. Haloarchaea have different metabolic adaptations to withstand these extreme conditions. Among the adaptations, several vesicles, granules, primary and secondary metabolites are produced that are highly significant in biotechnology, such as carotenoids, halocins, enzymes, and granules of polyhydroxyalkanoates (PHAs). Among halophilic enzymes, reductases play a significant role in the textile industry and the degradation of hydrocarbon compounds. Enzymes like dehydrogenases, glycosyl hydrolases, lipases, esterases, and proteases can also be used in several industrial procedures. More recently, several studies stated that carotenoids, gas vacuoles, and liposomes produced by haloarchaea have specific applications in medicine and pharmacy. Additionally, the production of biodegradable and biocompatible polymers by haloarchaea to store carbon makes them potent candidates to be used as cell factories in the industrial production of bioplastics. Furthermore, some haloarchaeal species can synthesize nanoparticles during heavy metal detoxification, thus shedding light on a new approach to producing nanoparticles on a large scale. Recent studies also highlight that exopolysaccharides from haloarchaea can bind the SARS-CoV-2 spike protein. This review explores the potential of haloarchaea in the industry and biotechnology as cellular factories to upscale the production of diverse bioactive compounds.

## 1. Introduction: Haloarchaeal diversity and ecology

**FIGURE 1:**
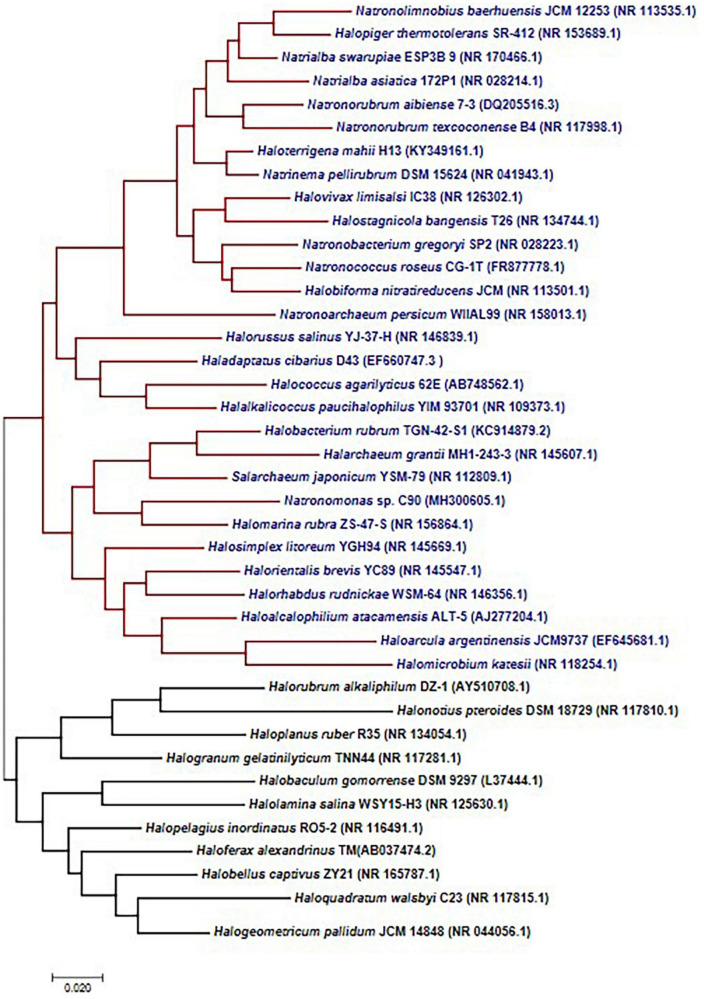
Haloarchaeal 16s RNA gene diversity. Phylogenetic tree constructed using the maximum likelihood methods suggests two separate clades, and most of the organisms are represented under a single clade.

Microorganisms possess several mechanisms to acclimatize to stress conditions that influence growth and survival in saline environments. Halophiles are microbes that can survive such saline conditions from low to high saturation points. There are different stress proteins and strategies that halophiles adapt to counteract stressful factors such as ions, temperature, pH, and UV radiation. Prokaryotic halophiles have attracted the attention of researchers worldwide because of their distinctive features, ease of manipulation, lesser space requirements for cultivation, and the production of diverse metabolites compared to plants or eukaryotic algae (Torregrosa-Crespo et al., 2017; Dutta and Bandopadhyay, 2022). A significant group of halophilic archaea, represented under the halobacteria class, are tolerant to extreme saline environments. These environments include salt lakes, estuaries, rivers, mangrove swamps, open seawater, coastal waters, salt lakes, estuaries, and salt deserts. Halobacterial class constitutes a wide range of genera (Figure 1) – *Salarchaeum, Halobiforma, Natronolimnobius, Halopelagius, Halogranum, Halonotius, Haladaptatus, Natronococcus, Haloferax, Halococcus, Haloalcalophilium, Halorubrum, Halorhabdus, Halorussus, Halopiger, Halomarina, Natronoarchaeum, Halobellus*, *Natrialba Halobaculum, Haloplanus, Halostagnicola, Halorientalis, Natronomonas, Natrialba, Natronobacterium, Natronorubrum, Haloarcula, Halobacterium, Haloterrigena, Natrinema, Halogeometricum, Halalkalicoccus, Haloquadratum, Halogeometricum, Natronorubrum, Halomicrobium, Halolamina, Halovivax, Halarchaeum*, and *Halosimplex* (Cui and Dyall-Smith, 2021).

Considering the difficulties found by researchers to obtain pure cultures of extremophilic microorganisms (including halophilic archaea) from environmental samples, and consequently to know the microbial biodiversity in those samples, recent advances in omic-based approaches have contributed to a better understanding of haloarchaeal biodiversity. Particularly, metagenomic analysis has been used to overcome this limitation. Metagenomic analysis of environmental samples from the Dead Sea, RedSea, Gulf of Cambay, Mediterranean Sea, Sundarbans mangrove forest, Karak Salt Mine, and Pannonian Steppe, revealed the predominance of haloarchaeal genera in the natural environment, including *Haloarcula*, *Halorubrum*, and *Halorhabdus* (Oh et al., 2010; Rhodes et al., 2012; Somboonna et al., 2012; Fernández et al., 2014; Bhattacharyya et al., 2015; Keshri et al., 2015; Behzad et al., 2016; Szabó et al., 2017; Haldar and Nazareth, 2018; Osman et al., 2019; Cecil et al., 2020). Similarly, a global metagenomic meta-analysis revealed the dominance of *Haloarcula* and *Haloquandratum* sp., in the seacoast (Moopantakath et al., 2021). The biogeography of haloarchaea also varies based on the biosystems. For instance, the *Haloferax* genus is highly dominant in seashores and island samples and estuaries, while the *Natrialba* genus is predominant in rivers, mangroves, and lakes (Hegazy et al., 2020; Moopantakath et al., 2020; Cho et al., 2021). Thus, the prevalence of halophilic microorganisms in general, and particularly of haloarchaea, is highly dependent on the environment, geography, and physicochemical parameters like salinity, pH, oxygen availability and sun radiation, among other factors (Ventosa, 2006; Mani et al., 2020). As an example, recent whole genome sequencing studies revealed that autotrophic haloarchaea are highly abundant in coastal environments, probably due to salinity and direct sunlight exposure (Moopantakath et al., 2021). These environments display unique features and salt deposition phenomena that contribute to the increase of some haloarchaeal populations requiring extremely high concentrations for optimal growth.

Environmental degradation, such as the release of industrial chemicals into the coastal environment, is also a major factor in pollution and shoreline contamination (Lu et al., 2018) and in microbial biodiversity. Nonetheless, the microbial community plays a significant role in the polluted site, and the unique metabolic features of haloarchaea contribute to the homeostasis of these environments. *Halorhabdus* and *Natrinema* sp., have been reported to degrade xylan and produce halocin (antimicrobial peptide), respectively (Begemann et al., 2011; Besse et al., 2017). Similarly, recent advances in the metagenomic analysis have revealed that haloarchaea, such as *Haloferax*, *Haladaptatus*, *Natrialba*, etc., participate in maintaining the biogeochemical processes in coastal ecosystems (Osman et al., 2019). Some haloarchaea can carry out interesting metabolic processes from an industrial point of view; examples: sulfur reduction by *Natranaeroarchaeum sulfidigenes* (Sorokin et al., 2022), nitrification by *Haloarcula, Halolamina*, and *Halobacterium* (Wei et al., 2022), phosphorus solubilization by *Haloarcula*, *Halobacterium, Halococcus*, and *Haloferax* (Yadav et al., 2015) or denitrification by *Haloarcula* or *Haloferax* (Miralles-Robledillo et al., 2021).

In summary, haloarchaea exhibit diverse metabolic pathways and biological activities of interest for biotechnological purposes. They constitute predominant microbial communities in salty ecosystems, which are widespread worldwide. For example, *Haloarcula*, *Haloferax*, *and Halogeometricum* sp., can be isolated from saline sediments across the world, such as from Spanish coastal and inland salted ponds (Martínez et al., 2022), Algerian salt lakes, Indian salt pans, Verkhnekamsk deposit etc., and exhibit several biological activities, including the production of carotenoid pigments (Das et al., 2019; Sahli et al., 2022). Besides, many more ecosystems characterized by their high salt concentrations are far from known from a microbiological point of view (i.e., saline mines in Senegal). This review aims to summarize new advances in the knowledge of biological applications of halophilic archaea and the synthesis of secondary metabolites thus contributing to the design of new biotech processes low cost and environmentally friendly based on the use of haloarchaea as cellular factories.

### 1.1. Haloarchaeal strategies to cope with stress

Haloarchaea can survive in stress conditions such as salinity, ultraviolet (UV), high concentration of ions, high temperature, and extreme pH values. In addition, continuous heavy rain or change in temperature can lead to a dramatic shift in the salinity, causing significant pressure on haloarchaea and promoting the switching on of molecular machinery to be better adapted to these environmental changes (Griffiths and Philippot, 2013; Thombre et al., 2016). Halophiles inhabiting saline environments can exist at different salt concentrations, mainly above 1 M. Based on the optimum salinity requirements, halophiles can be classified into slight halophiles (0.34 to 0.85 M), moderate halophiles (0.85 to 3.4 M), and extreme halophiles (3.4 M to saturation point) (Abaramak et al., 2020). Haloarchaea requires ∼10 to 35% w/v (1.71 to 6 M) of salt concentration for optimum growth. Interestingly, haloarchaea are the dominant class of microbes when the salt concentration increases above 16% w/v (2.74 M) (Oren, 2002b; Andrei et al., 2012).

Haloarchaea evolved with several metabolic adaptations to survive different stresses among which salt stress is one of the most significant affecting the protein structures and therefore, their biological activities (Britton et al., 2006). Thus, most of their proteins are salt dependent for optimal enzymatic activity and stability. The unique feature of these proteins is due to the presence of acidic amino acids on the surface (Esclapez et al., 2007). The negatively charged acidic amino acids on the surface develop into a cluster form and interact with networks of hydrated ions. Consequently, this feature avoids the precipitation of haloarchaeal proteins under high KCl/NaCl concentrations. Also, proteins exhibit less hydrophobic interactions owing to the limited content of hydrophilic amino acids such as lysine. Hence, a lack of optimum salt concentration may cause the unfolding of proteins owing to the presence of negatively charged amino acids (Kennedy et al., 2001; Oren, 2002a; Zafrilla et al., 2010; Andrei et al., 2012).

Some haloarchaea are called polyextremophiles owing to their ability to respond to multiple extreme conditions (Das and Dash, 2018). They can adjust to osmotic stress and survive at low water activity and desiccation. The presence of high salt concentrations may reduce water activity from 1 to 0.75. Further, salt-in and low-salt-in are the two methods adopted by haloarchaea to resist osmotic stress. In the salt-in method, K^+^ is accumulated inside cells with the help of protein transport and ion pumps (Schäfer et al., 1996; Oren, 2013). *Halobacterium* sp., NRC-1 utilizes the salt-in strategy with the help of potassium transporters and sodium efflux pumps (Ng et al., 2000).

In the low-salt-in method, some organisms produce compatible and low molecular-weight solutes to adapt to osmotic stress (Grant et al., 2004). The compatible solutes such as amino acids, ectoines, thetines, polyols, betaines, derivatives of sugar, and glutamine amide are accumulated in low concentration in the cytoplasm to tolerate osmotic stress (Matarredona et al., 2020). For instance, solutes like 2-sulphotrehalose, and glycine produced by *Natronobacterium* and *Natronococcus*, respectively, help in the low-salt process (Grant et al., 2004; Matarredona et al., 2020). The square-shaped haloarchaea, *Haloquadratum walsbyi*, carries a unique protein, halomucin, which helps to survive desiccation. The protein is glycosylated and sulfated to develop as a water-rich capsule around the archaea. The water-rich cloud formed around the cell protects it from surrounding desiccation or the presence of low water activity (Bolhuis et al., 2006; Dyall-Smith et al., 2011; Zenke et al., 2015).

Regarding stress due to temperature changes, haloarchaea can tolerate different temperature variations in saline environments thanks to the presence of heat shock proteins (e.g., chaperones and chaperonins). The molecular chaperones are involved in the folding or unfolding of proteins at extreme temperatures (Fenderson, 2006; Shukla, 2006; Coker et al., 2007). The most common heat shock proteins observed in haloarchaea are Hsp60 and 70 (Macario et al., 1999). The expression and synthesis of cold shock proteins, polar lipids, and gas vesicles in cold temperatures help to maintain homeostasis (Coker et al., 2007). Some haloarchaea, for example, *Haloferax*, produce thermoprotectans, such as glycoside hydrolases, to withstand high temperatures by promoting protein stabilization (Amin et al., 2021).

The ecosystems inhabited by haloarchaea are exposed to high sun radiation doses that cause UV irradiation and the formation of photoproducts and pyrimidine dimers in DNA. The photoreactivation process can remove these lesions with the help of photolyase expressed by haloarchaea. Haloarchaea has unique compounds that include rhodopsin which has a phototaxis mechanism. On the other hand, gas vesicles play a crucial role in light regulation and responses to oxygen availability changes (Jones and Baxter, 2017; Miralles-Robledillo et al., 2021). Response to UV irradiation also includes the downregulation of genes involved in the gas vesicle production to sink the cells below the water surface (Kottemann et al., 2005). Some haloarchaea, such as *Halorubrum lacusprofundi* and *Haloferax volcanii*, can withstand a wide range of pH. They can exist in both low-pH environments like acidic lakes and alkaline lakes (Mormile et al., 2009). In the case of *Halorubrum lacusprofundi*, *Haloferax volcanii*, and *Halobacterium* sp., residing in alkaline pH conditions, it has been described that several stress genes such as *hsp20* family, universal stress protein *uspA*, or *groEL* chaperone are upregulated. In contrast, *H. lacusprofundi* exhibited upregulation of *hlac3059* and *hlac3556* gene expression in acidic pH. Besides, they display dormancy-specific responses at acidic pH to survive in the environment (Moran-Reyna and Coker, 2014).

## 2. Biotechnological significance of haloarchaea

Several biotechnological-based processes can be benefited from the use of haloarchaea. Thus, the use of whole cells in wastewater treatments and bioremediation of brines and salty soils has been recently revealed as a promising tool. Hypersaline wastewater is a common byproduct of industrial processes. Hence, cost-effective treatment of hypersaline wastewater is necessary for sustainable development. Biological wastewater treatment has been considered a more economical approach, but mesophilic microorganisms used so far for this purpose can not be used in the biological treatment of polluted brines of wastewater containing high salt concentration. In this context, recent research findings revealed the role of haloarchaea in the treatment of salty wastewater and brine generated as final residues in water desalination plants (Li et al., 2021; Martínez et al., 2022). Haloarchaea can also degrade hydrocarbons; however, their degradation is more efficient for low molecular-weight hydrocarbons. The degradation of naphthalene, phenol, p-hydroxybenzoic acid, and 3-phenyl propionic acid, and oxychlorides like perchlorate or chlorate by haloarchaea also make it an attractive choice for wastewater treatment (Martínez-Espinosa et al., 2015; Mukherji et al., 2020; Li et al., 2021). Another interesting approach related to wastewater treatments is the removal of nitrogen to avoid eutrophication in the receiving water bodies and heavy metals to avoid global toxicity. Haloarchaea, such as *Haloferax mediterranei*, can use NO_3_^–^ and NO_2_^–^ as nitrogen sources for growth or as final electron acceptors instead of oxygen in an anaerobic respiratory process (denitrification). For example, *Haloferax mediterranei* encodes nitrate reductase (*nas*) and nitrite reductase (*nir*) that can perform assimilatory nitrate/nitrite reduction (Martínez-Espinosa et al., 2007), whilst Nar, NirK, Nor, and Nos encodes key enzymes in catalyzing the reactions involved in the process of denitrification (Bernabeu et al., 2021). Regarding heavy metals, several recent studies have demonstrated that some haloarchaeal species can grow in the presence of heavy metals at concentrations that are toxic for most living beings. In some cases, because of cellular growth, heavy metals are accumulated, modified or bioassimilated. Molecular machinery for the potential removal of cooper and cadmium has also been identified from many haloarchaeal species (Vera-Bernal and Martínez-Espinosa, 2021; Llorca and Martínez-Espinosa, 2022). In other cases, haloarchaea growing in the presence of heavy metals can also synthesize nanoparticles (NPs) from wastewater polluted with cadmium, arsenic, and zinc (Taran et al., 2017; Li et al., 2021; Gaonkar and Furtado, 2022). Haloarchaea have various mechanisms to tolerate the arsenate metals using minichromosomes/megaplasmids (*arsADRC* gene cluster) in the *Halobacterium* species (Wang et al., 2004; Voica et al., 2016). The zinc tolerance mechanism was found due to the presence of physical bioabsorption, ion exchange and intracellular accumulation which can be used for the various biological process inside the cell (Popescu and Dumitru, 2009; Williams et al., 2013; Salgaonkar et al., 2016). Similarly, cadmium also plays a crucial aid in intracellular metabolic functions so cadmium can be tolerated inside the cell without stress (Vera-Bernal and Martínez-Espinosa, 2021).

Another interesting biotechnological application is related to the enzymes from haloarchaea. They exhibit increased tolerance, not only to salinity but also to pH, pressure, temperature, etc. Haloenzymes have several advantages, such as minimum steps in purification, sterilization, and cost-effectiveness (Amoozegar et al., 2017). Haloenzymes such as lipase and alcohol dehydrogenase from *Haloferax volcanii* and *Haloarcula* sp., G41, respectively, have been immobilized successfully for increased activity (Alsafadi and Paradisi, 2014; Li and Yu, 2014). Saline-tolerant lipases and esterases like those described from *Haloarcula marismortui* and *Natronococcus* sp., TC6 are essential enzymes in several biotechnological applications such as biofuel, detergent, textile, etc (Camacho et al., 2009; Del Campo et al., 2015).

**FIGURE 2:**
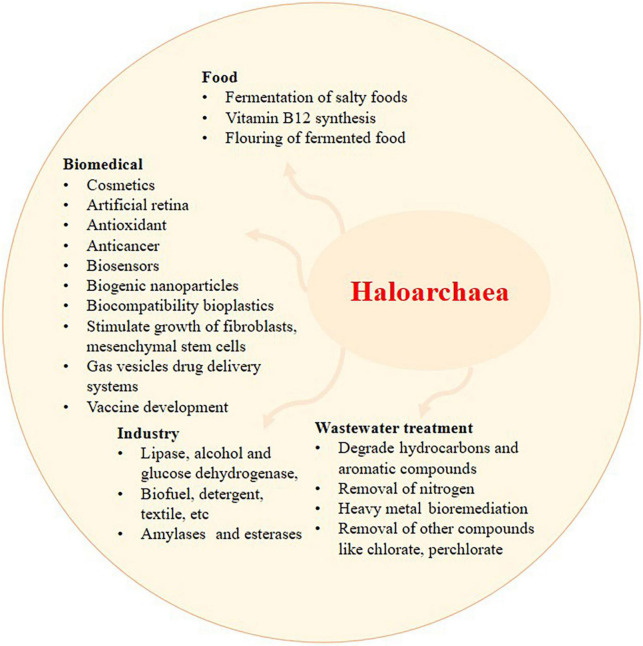
The application of haloarchaea and its metabolites. Recent exploration of haloarchaea and its metabolites has shed light on their potential applications in wastewater treatment, biomedical, food, and industrial sectors. Haloarchaea is a preferred source of haloenzymes since the growth and purification of enzymes involve minimal steps and are prone to less contamination.

The haloarchaeal membrane protein bacteriorhodopsin, initially discovered from *Halobacterium salinarum*, is highly stable to thermal and photochemical stress. Bacteriorhodopsin can sense light and convert it into electrical signals (Singh and Singh, 2017). Bacteriorhodopsin from haloarchaea has also found applications in biosensors and artificial retinas (Figure 2; Amoozegar et al., 2017). The gas vesicles mentioned in section “1. Introduction: haloarchaeal diversity and ecology” have applications in drug delivery systems and vaccine development (Cánovas et al., 2021). Meanwhile, poly-β-hydroxy-alkanoates (PHAs) produced by haloarchaea, such as *Haloferax mediterranei*, are considered an alternative to plastics produced from petroleum and in medical applications owing to their biocompatibility (Salgaonkar et al., 2013; Li et al., 2021). Thus, PHA can be synthesized using these microorganisms as biofactories thanks to cheaper procedures in which the biomass downstream process as well as the purification process of the biopolymer can be done with single steps (Simó-Cabrera et al., 2021). PHA produced by various materials such as residues from food and agriculture products can be synthesized into PHA with a help of haloarchaea (Quillaguamán et al., 2010). Genomic insights of halophiles such as *Halonotius terrestris* sp., nov. and *Halonotius roseus* sp., nov. have been recently delineated and found to encode the complete biosynthetic genes for the biosynthesis of cobalamin (vitamin B12) (Durán-Viseras et al., 2019).

Halophiles produce saline-tolerant proteins and metabolites, such as carotenoids, which assist in tolerance toward salinity. Haloarchaeal carotenoids have industrial interest due to their antioxidant, anticancer, antimicrobial, anti-inflammatory, food colorant, and several other biomedical applications (Verma et al., 2020). Bacterioruberin is also commonly used in cosmetics and drug encapsulation (Serrano et al., 2022). Haloarchaea have been extensively explored for bioactive metabolites with anticancer, antimicrobial, and antioxidant activities (Table 1). Among the several haloarchaeal secondary metabolites, carotenoids gained attention due to their multi-faced applications in cosmetics, food, and biomedical sectors (Serrano et al., 2022). Carotenoids have multiple roles in bacteria, plants, and archaea. Carotenoid helps photosynthetic plants and microbes to increase light absorption in the blue-green region through the singlet-singlet energy transfer (Hashimoto et al., 2015). It also has photoprotective effects against excessive light and reactive oxygen species through the triplet energy transfer of chlorophylls to carotenoids (Maoka, 2020). They are crucial in structural stabilization and render tolerance to hypersalinity.

**TABLE 1:** Overview of the biological application of haloarchaea and their compounds: anticancer, antioxidant and antimicrobial activities of the metabolites derived from haloarchaea.

Haloarchaea	Compounds	Complementary information	References
**Anticancer activity**			
*Halobacterium halobium*	Carotenoid	Antiproliferative activities (>0.5 μm)	Abbes et al., 2013
*Halogeometricum. limi*	Carotenoid	HepG2 cells, 23% at a high concentration of 720 μg/L (∼1 μm)	Hou and Cui, 2018
*Halobiforma* sp.,	Superparamagnetic iron oxide nanoparticles	Localized hyperthermia cancer therapy	Salem et al., 2020
*Haloferax mediterranei*	Carotenoid	HER2-positive or triple-negative breast cancer (TNBC)	Giani and Martínez-Espinosa, 2020
*Natrialba* sp., M6	Carotenoid	Normal human lung fibroblast cells (Wi-38) 50 and 100% cell viability.	Hegazy et al., 2020
		50% cell death for Caco-2 (colon cancer line),	
		MCF7 (breast cancer cell line)	
		HepG2 (liver cancer line)	
		HeLa (cervical cancer cell line)	
**Antioxidant activities**			
*Haloferax*	Carotenoid	DPPH:IC_50_ = 56.69 μg/ml,	Sahli et al., 2022
		ABTS:IC_50_ = 39.66 μg/ml	
*Halogeometricum*	Carotenoid	DPPH:IC_50_ = 170.4 μg/ml,	
		ABTS:IC_50_ = 136.43 μg/ml	
Genetically modified *Haloferax volcanii* strain (HVLON3)	Bacterioruberin	EC_50_ yielded 4.5 × 10^–5^ mol/l	Zalazar et al., 2019
*H. Hispanica* hm1	Carotenoid	ABTS (88%; IC_50_ = 3.89 μg/ml), FRP assay (82%; EC_50_ = 3.12 μg/ml)	Gómez-Villegas et al., 2020
Hfx. Volcanic, Hgn. rubrum, and Hpl. coordinates	Carotenoid	DPPH radical scavenging activity > 80% at 10 ug/ml	Hou and Cui, 2018
*Haloferax mediterranei*	Carotenoid	Oxidative stress	Giani et al., 2021
*Haloferax mediterranei*	Carotenoid	Antioxidant, antiglycemic, and antilipidemic activities	Giani et al., 2022
**Antimicrobial activities**			
*Halogeometricum* sp., ME3, *Haloarcula* sp., BT9, *Haloferax* sp., ME16	Carotenoid	*Vibrio anguillarum*, *Pseudomonas aeruginosa*, *Pseudomonas anguilliseptica*	Sahli et al., 2022
*Haloferax alexandrinus*	AgNPS	*Pseudomonas aeruginosa* ATCC 9027, *Bordetellabronchiseptica* ATCC 4617, *Staphylococcusaureus* ATCC 6538P	Patil et al., 2014
*Haloferax alexandrinus* and *Haloferax lucentense*	AgCl-NPS	*Pseudomonas aeruginosa* and *Bacillus* sp	Moopantakath et al., 2022

Due to the advancements in culture-based methods and metagenomics, the identification and discovery of novel species of haloarchaea become a continuous process. Recently, 12 haloarchaeal isolates were isolated from Tamil Nadu (India), out of which nine isolates were novel. Interestingly, all of the isolates produced carotenoids (Verma et al., 2020). Haloarchaea has an inherent molecule known as bacterioruberin, which is found in most haloarchaea (Nagar et al., 2022). Similarly, novel species under the genus *Halorubrum*, isolated from South Korea, produced C_50_ carotenoid (bacterioruberin), having a strong antioxidant activity (Hwang et al., 2022). Likewise, carotenoids from novel *Haloarcula* sp., and *Halorubrum* sp., strains were isolated from the Atacama Desert (Lizama et al., 2021). Large-scale whole genome (*n* = 68) analysis suggested that haloarchaea has a wide diversity of carotenoid biosynthetic genes (Serrano et al., 2022), suggesting that haloarchaeal species could be a reservoir of carotenoid derivatives.

### 2.1. Anticancer compounds

Haloarchaea and their metabolic products are getting more attention for the treatment of several cancers. Carotenoid pigment from *Halobacterium halobium*, isolated from a saltern in Tunisia, exhibited anticancer activity against the HepG2 cell line (Abbes et al., 2013). In addition, the carotenoid pigment of *Halogeometricum limi*, at a concentration of 720 μg/l, exhibited ∼23% anticancer activity against HepG2 cells (Hou and Cui, 2018). Similarly, high carotenoid-producing (0.98 g/l) haloarchaea i.e., *Natrialba* sp., M6, which thrives at 25% NaCl and a pH of 10.0, exhibited 50% anticancer activity against MCF-7, HepG2, and HeLa cells at low concentrations (<39 μg/ml) (Hegazy et al., 2020).

Compared to β-carotene, bacterioruberin has a superior antihemolytic and cytotoxic effect against HepG2 cells (Hou and Cui, 2018). *Natrialba* sp., M6, under the phylum Euryarchaeota, isolated from Egypt, produced C_50_ carotenoid as the predominant compound (Hegazy et al., 2020). The pigment had a higher selectivity toward cancer cells than the standard chemotherapeutic agent 5-fluorouracil. It is active against breast, liver, and colon cancer cells. On the other hand, C_50_ carotenoid bacterioruberin with dexamethasone reduced the release of TNF-α and IL-8, reversed the inflammation-induced morphological changes of macrophage, and had a potential role as an intestinal barrier repairing agent (Higa et al., 2020).

### 2.2. Antimicrobial compounds

Microorganisms causing infectious diseases evolve and acquire antimicrobial resistance continuously. Thus, alternative antimicrobials are required to meet emerging health challenges. Haloarchaeal carotenoids as antimicrobials have been explored less compared to other carotenoids. However, the potential of haloarchaeal carotenoids as antimicrobials was demonstrated against several pathogens (Gómez-Villegas et al., 2020). Recent reports have shown that carotenoids from *Halogeometricum* sp., ME3, *Haloarcula* sp., BT9 and *Haloferax* sp., ME16 have antimicrobial activity against *Vibrio anguillarum*, *Pseudomonas aeruginosa*, and *Pseudomonas anguilliseptica*, respectively (Sahli et al., 2022).

Haloarchaea produce halocins with potent antimicrobial activity. Halocin from the supernatant of *Haloferax larsenii* HA1 exhibits potent antimicrobial activity (Kumar and Tiwari, 2017). Previous works on halocines from haloarchaea suggested that the antimicrobial action of halocins could be related to processes of competition between different species for niche, food, or space. For instance, *Halobacterium salinarum* ETD5, isolated from the solar saltern of Sfax, Tunisia, exhibited antagonistic activity against haloarchaea of similar niches such as *Halorubrum* sp (strain ETD1, ETD2, ETD6, ETR7), *Halorubrum chaoviator* sp (strains ETD3, ETR14, and SS1R17), and *Halobacterium salinarum* ETD19 (Ghanmi et al., 2016). Furthermore, the C50 carotenoid pigment from *Natrialba* sp., M6 also exhibited promising potential in the elimination of hepatitis C virus (HCV) and hepatitis B virus (HBV) from human blood mononuclear cells suggesting its strong antiviral activity (Hegazy et al., 2020).

### 2.3. Antioxidant compounds

The human body produces free radicals during metabolic processes, which create oxidative stress and contribute to inflammation and lifestyle diseases. At present, haloarchaeal compounds have received significant attention due to their free radical scavenging properties at lower concentrations which are considerably more effective than the standard reference compounds like ascorbic acids. Bacterioruberin has a conjugated structure containing 13 C-C units and has a high free radical scavenging activity. The carotenoid from *Haloferax* sp., exhibited high antioxidative activity, confirmed with DPPH (2,2-diphenylpicrylhydrazyl) and ABTS (2,2’-azino-bis(3-ethylbenzothiazoline-6-sulfonic acid) assay. The IC_50_ values for the carotenoid compound were 56.69 and 39.66 μg/ml in DPPH and ABTS assay, respectively (Sahli et al., 2022). In contrast, carotenoid pigments isolated from the *Halogeometricum* sp., exhibited antioxidative activity with an IC_50_?170.4 μg/ml (DPPH assay) (Sahli et al., 2022). Similarly, acetone extracts from the *H. hispanica* HM1 showed 88% (ABTS) and 82% (Ferric ion reducing power) activity (Gómez-Villegas et al., 2020). Similarly, carotenoids from *H. volcanii*, *Hgn. rubrum*, and *Hpl. Inordinat*ehave highlighted higher (80%) free radical scavenging properties at a concentration of 10 μg/ml (Hou and Cui, 2018). Zalazar et al. (2019) reported a genetically modified *Haloferax volcanii* strain (HVLON3) with high antioxidative activities (EC_50_ = 4.5 × 10^–5^ mol/l). Thus, haloarchaeal compounds can serve as a prominent source of antioxidant molecules in the future.

Bacterioruberin and β-carotene are the most desirable carotenoids for biological applications. Bacterioruberin, as an antioxidant molecule, can capture reactive oxygen species. Its exhibits antioxidant activity higher than the standard ascorbic acid (Sahli et al., 2022). Bacterioruberin has superior antioxidant properties compared to the β-carotene (Zalazar et al., 2019). Oxidative stress in the form of H_2_O_2_ increases the bacterioruberin production in *Haloferax mediterranei* strain R-4 up to 78% (Giani and Martínez-Espinosa, 2020). Furthermore, the antioxidant activity of haloarchaea varies between species. For instance, carotenoids from *Haloferax* sp., ME16 have a higher antioxidant activity than *Halogeometricum* and *Haloarcula* (Sahli et al., 2022).

## 3. Hydrolytic enzymes from haloarchaea

The wide range of enzymes produced by halophiles plays a vital role in biotechnology, including biosynthesis, food processing industries, and bioremediation methods. As the enzymes produced by haloarchaea are stable at high salt concentrations they can be utilized in several processes related to food or leather tanning. The most important hydrolytic enzymes produced by haloarchaea are proteases and lipases (Modern et al., 2000). The first hydrolase enzyme, a serine protease, was purified and studied from *Halobacterium salinarum*. This enzyme was active only at a high concentration of NaCl (more than 2 M), being an enzyme rich in negatively charged amino acids (Ventosa et al., 2005). Starch-degrading alpha-amylases are synthesized by halophiles such as *Halobacterium salinarum*, *Haloferax mediterranei*, *Halomonas meridiana*, and *Natronococcus amylolyticus* (Kumar et al., 2016). Besides amylases, pullulanase is produced by different archaea, such as *Halorubrum* sp., Ha25, which is already used in the starch industry (Siroosi et al., 2014). Cellulase-degrading cellulases and glycoside hydrolase gene homologs are present in *Halorhabdus utahensis* (Zhang et al., 2011), *Haloarcula* sp., (Ogan et al., 2012), *Halorubrum lacusprofundi* (Karan et al., 2013), *Haloarcula vallismortis* (Nercessian et al., 2015), *Natronobiforma cellulositropha* (Sorokin et al., 2018), *Halalkalicoccus jeotgali* (Anderson et al., 2011), and *Haloferax sulfurifontis* (Malik and Furtado, 2019). Table 2 displays different intracellular or extracellular hydrolytic enzymes produced by haloarchaea.

**TABLE 2:** Hydrolytic enzymes produced by haloarchaea and their biosynthesis mode.

Haloarchaea	Hydrolytic enzymes	Location	References
*Haloterrigenaturkmenica*	α-amylase	Extracellular	Santorelli et al., 2016
*Haloferax mediterranei*	Monomeric α-amylase	Extracellular	Pérez-Pomares et al., 2003
*Haloarcula* sp., strain S-1	Organic solvent tolerant α-amylase	Extracellular	Fukushima et al., 2005
*Haloarculahispanica*	Highly stable α-amylase	Extracellular	Hutcheon et al., 2005
*Haloarcula* sp., HS	Poly-extremotolerant α-amylase	Intracellular and extracellular	Gómez-Villegas et al., 2021
*Haloarcula* sp., LLSG7	Organic solvent-tolerant cellulase	Extracellular	Li and Yu, 2013
*Halorhabdus utahensis*	Heat and ionic liquid-tolerant cellulase	Extracellular	Zhang et al., 2011
*Halomicrobium* and *Salinarchaeum*	Chitinase	Extracellular	Sorokin et al., 2015
*Haloterrigena*	Chitinase	Intracellular	Sorokin et al., 2015
*Halobacterium salinarum* NRC-1	Chitinase	Extracellular	Yatsunami et al., 2010
*Haloferax mediterranei* S1	Lipase	Extracellular	Akmoussi-Toumi et al., 2018
*Haloarcula* sp., G41	Organic solvent tolerant lipase	Extracellular	Li and Yu, 2014
*Natrialba asiatica* 172 P1	Protease	Extracellular	De Castro et al., 2006
*Halobacterium salinarum* I and IM	Protease	Extracellular	De Castro et al., 2006
*Natronococcus* sp., TC6	Esterase	Extracellular	Martin del Campo et al., 2015
*H. marismortui*	Esterase and lipase	Intracellular	Camacho et al., 2009
*Haloferax volcanii*	Laccase	Extracellular	Uthandi et al., 2010

Amylase, one of the important industrial enzymes, was reported to synthesize by a new haloarchaeal strain isolated from salterns. High amylase activity was exhibited by the new strain *Haloarcula* sp., HS and found to be poly extremotolerant. The optimum enzyme yield was obtained at high salt concentrations (25%), 60°C, and was calcium-dependent. Amylases were found to be synthesized in extracellular and intracellular fractions and observed as 3 different types of enzymes. The extracted enzymes were tested on bakery waste. It was found that amylases degraded bakery waste efficiently at high salt concentrations (Gómez-Villegas et al., 2021). Interestingly, extracellular hydrolytic enzymes were produced by haloarchaeal strains obtained from hypersaline lakes. The two most abundant archaeal species, *Natrinema* and *Halorubrum*, produced cellulase, pectinase, amylase, lipase, and xylanase but not protease (Karray et al., 2018). In the study, alpha-amylase obtained from *H. salinarum* was immobilized in calcium alginate to enhance its stability (Patel et al., 1996). In another study, the tolerance of alcohol dehydrogenase enzyme, obtained from *Haloferax volcanii*, to organic solvents was reported to improve upon immobilization with sepabeads (Alsafadi and Paradisi, 2014). Immobilized lipase enzyme on anionic resin obtained from *Haloarcula* sp., was used to produce biodiesel (Li and Yu, 2014). *Halobacterium salinarum* NRC-1, *Haloarcula japonica*, *H. salinarum* CECT 395, and *H. mediterranei* can grow on chitin thanks to chitinases (García-Fraga et al., 2014; Hou et al., 2014). Haloarchaea degrades lignin through laccase and peroxidase enzymes. Some haloarchaea produces enzymes like esterases and lipases to degrade ester, ether, and glycosidic linkages. Menasria et al. (2018) have reported extensive bioprospecting of salt-stable and active hydrolytic enzymes from haloarchaea of arid and semi-arid wetlands. The major haloarchaea identified were from the class halobacteria such as *Haloarcula*, *Halogeometricum*, *Halococcus*, *Haloterrigena*, etc. Among the 68 isolates screened, 89.7% of isolates produced 2 halophilic enzymes, whereas 52.9% produced 3 hydrolytic enzymes. These isolates produced gelatinase, cellulase, esterase, and inulinase. Secondly, some isolates were profound in producing xylanase, pectinase, and nuclease. The study also reported that the high cellulase activity (35%) makes it a potential candidate in the food and textile industries (Menasria et al., 2018). Likewise, out of 300 isolates from a salt lake, 293 haloarchaea isolates were selected and studied for active hydrophilic hydrolytic enzymes.

The cellulase, xylanase, amylase, DNase, lipase, protease, pullulanase, chitinase, and inulinase were observed in 9 potential isolates. The most abundant enzymes produced by haloarchaeal isolates (*Halorubrum* and *Haloarcula*) were lipase, DNase, and amylase (Makhdoumi Kakhki et al., 2011).

## 4. Biodegradable and biocompatible polymers by haloarchaea

Search for eco-friendly biopolymers is one of the important research objectives worldwide to reduce global plastic pollution and in the production of biomedical devices (Simó-Cabrera et al., 2021). Regarding biomedicine, the desirable properties of biodegradable polymers vary based on their application, such as the high degradative potential for surgical mesh and low degradative potential for bioengineered skin. For such applications, biopolymers can be synthetically prepared, such as polylactic acid (PLA) and polyglycolic acid (PGA) (Han et al., 2015). Yet such synthetically prepared biopolymers are associated with setbacks such as biocompatibility and inflammation. Among natural biopolymers with interesting physicochemical properties to be used as bioplastic for packaging or biomedical applications, polyhydroxyalkanoates (PHAs) from haloarchaea are of research interest. The advantages of PHAs include their ease of mechanical customization, biodegradability, and biocompatibility (Han et al., 2015). Hence, PHAs have been under extensive research for application in medical implants, drug delivery, tissue replacement, etc. PHAs, composed of hydroxyalkanoate monomers, are stored as a source of carbon and energy under stress conditions in archaea and bacteria. The type of hydroxyalkanoate monomers determines the physical properties (rigid or elastic) of the PHAs. Among the PHAs, the most common ones are poly-3-hydroxybutyrate (PHB) and poly(3-hydroxybutyrate-co-3-hydroxyvalerate) (PHBHV). PHBHV comprises PHB and 3-hydroxyvalerate (3HV) monomer. PHBHV, upon degradation in the body, does not release toxic byproducts, has more outstanding biocompatibility and biodegradation, and helps in the growth of fibroblasts, mesenchymal stem cells, etc (Ahmed et al., 2010).

Archaea are considered promising cell factories and more cost-effective than bacteria for PHBHV production. For instance, *Haloferax mediterranei* produces PHBHV with a lower melting point than *Hydrogenophaga pseudoflava* (Koller et al., 2007). PHBHV produced by *Halogranum amylolyticum* has a higher hemocompatibility than *Ralstonia eutropha* (Zhao Y. et al., 2015). Furthermore, the entire process of biosynthesis is within the cell. PHAs are water-insoluble, degradable without oxygen, and increase their solubility in chlorinated solvents (Simó-Cabrera et al., 2021). Based on the monomers, PHAs are classified as homopolymers consisting of the same type of monomers (P4HB, P3HP, P3H4P, etc.), random copolymers consisting of multiple monomer types distributed random, and block copolymers where the distinct polymers are distributed in discrete blocks (Simó-Cabrera et al., 2021). *Haloferax mediterranei* can also produce varying types of PHBHV based on the concentration of valerate being fed (Han et al., 2015). These variations are of two main types, higher-order copolymers (O-PHBHV) composed of PHB and PHV with random PHBHV segments. R-PHBHV comprises random copolymers 3HB and 3HV (Han et al., 2015). PHAs are also produced by *Halogranum amylolyticum* TNN58 with poly (3-hydroxybutyrate-co-3-hydroxyvalerate) (PHBV) and 3-hydroxyvalerate (3HV) fraction when the carbon source is glucose (Zhao Y. X. et al., 2015). Genetic engineering, such as CRISPR-Cas technology, can enhance gene expression for PHAs production. A recent study showed a ∼165% increase in the output of PHA when *citZ* and *gltA* genes were downregulated by CRISPRi (Lin et al., 2021). Recently, robust methods have been developed for monitoring PHA granules in *Haloferax mediterranei*, such as through confocal fluorescence microscopy stained with Nile red and SYBR Green (Cánovas et al., 2021).

## 5. Synthesis and application of bioactive nanoparticles from haloarchaea

Nanobiotechnology is a boon to the field of medicine, which deals with the synthesis and application of a wide range of NPs for the treatment of diseases and targeted delivery of drugs (Patra et al., 2018; Dash et al., 2020). Synthesis of NPs using biological entities is always important due to their ease of production, eco-friendly approach, and bioavailability. The green approach in nanotechnology involves producing stable NPs capped with metabolites employed by organisms or plants (Singh et al., 2018). Several works have demonstrated that some haloarchaea can synthesize nanoparticles. However, the synthesis of NPs using haloarchaea needs to be better explored.

Recently, haloarchaea has been explored due to its ability to produce several NPs by detoxifying heavy metals. These organisms survive in the presence of heavy metals by employing enzymatic reduction of metals and sequestration methods to detoxify them (Voica et al., 2016). Table 3 shows different NPs synthesized using haloarchaea as well as their potential biological applications (Table 3). Additional factors such as salt, pH, temperature, and size affect the stability and efficiency of NPs (Dutta and Bandopadhyay, 2022).

**TABLE 3:** Different types of nanoparticles synthesized using haloarchaea and their biological activities.

Haloarchaea	Nanoparticles	Applications	References
*H. salifodinae* BK3 and BK6	Intracellular silver	Antibacterial activity (gram-positive and negative)	Tiquia-Arashiro and Rodrigues, 2016
*Haloferax* sp	Intracellular silver	Antibacterial activity against pathogenic bacteria	Abdollahnia et al., 2020
*Halogeometricum* sp	Intracellular selenium	Antibacterial activity against pathogenic bacteria	Abdollahnia et al., 2020
*Haloferax alexandrinus* RK_AK2 and *Haloferax lucentense* RK_MY6	Silver chloride	Anti-inflammatory, antioxidant, and antibacterial activity	Moopantakath et al., 2022
*Haloferax* sp., NRS1	Intracellular silver	Non-hemolytic (non-toxic) activity	Tag et al., 2021
*Halobacterium* sp., NRC-1	Self-adjuvant gas vesicle nanoparticles	Antigen delivery and development of salmonella vaccines	DasSarma et al., 2015
*Halomonas elongata* IBRCM	Zinc oxide	Antibacterial activity (*E. coli* and methicillin-resistant *S. aureus*)	Taran et al., 2018
*Halococcussalifodinae* BK3	Needle shape tellurium NPs	Antibacterial activity (gram-positive and negative)	Srivastava et al., 2015
*Halobiforma* sp., N1	Superparamagnetic iron oxide NPs	Hyperthermia treatment of cancer	Salem et al., 2020

Abdollahnia et al. (2020) studied the synthesis of silver and selenium nanoparticles by haloarchaea isolated from solar salterns. Intracellular production of silver and selenium NPs were reported by *Haloferax* sp., and *Halogeometricum* sp., respectively (Abdollahnia et al., 2020; Nagar et al., 2022). The biosynthesized nanoparticles exhibited antibacterial activity against *S. aureus, E. coli*, *B*. *subtilis*, and *P. aeruginosa* (Abdollahnia et al., 2020). A haloarchaea, *Haloferax* sp., NRS1 screened from solar saltern found in Saudi Arabia showed promising potential in synthesizing silver NPs. The biogenic silver NPs showed non-hemolytic activity below 12.5 μg/ml concentration suggesting their thrombolysis property. The non-toxic/hemolytic property of synthesized NPs potentiates their application as nano drug carriers (Tag et al., 2021).

Similarly, silver NPs synthesized by another group exhibited broad antimicrobial activity. The archaea *Halococcus salifodinae* BK6, mediated synthesis of silver NPs, employs NADH-dependent nitro reductases to reduce metal to nanoparticles. They showed promising antibacterial activity against *S. aureus* and *M. luteus* (Gram-positive) and *E. coli* and *P. aeruginosa* (Gram-negative) (Srivastava et al., 2014b).

Srivastava et al. (2014a) discussed the synthesis of selenium NPs using *H. salifodinae* BK18. Similar to their previous work, nitro reductases reduced sodium selenite to NPs. The intracellular synthesized NPs exhibited antiproliferative properties against HeLa cancer cell lines. Also, the NPs were found to be non-toxic against normal cells suggesting their application as an anticancer agent (Srivastava et al., 2014a). Bioactive gold and silver NPs were synthesized using *Haloferax volcanii*. Significant antibacterial activities were observed with synthesized silver NPs against *E. coli* and *P. putida* (Costa et al., 2020). In addition, haloarchaeal nanoparticles hold promising antibacterial properties. *Haloferax alexandrinus* was used to synthesize silver nanoparticles with an average size of 18 nm and with an amide carbonyl group on the surface. This synthesized nanoparticle has shown antimicrobial activity against *P. aeruginosa, Bordetella bronchiseptica*, and *S. aureus* at 5 μg concentrations (Patil et al., 2014). Another study also highlights the synthesis of spherical-shaped silver chloride nanoparticles (MY6-NP and AK2-NPs) with a size of 30–50 nm using *Haloferax Alexandrinus* and *Haloferax lucentense* at high salt-saturated conditions. According to a study by Salem et al. (2020), hyperthermia therapy can be employed with the help of superparamagnetic iron oxide nanoparticles synthesized using *Halobiforma* sp., N1. The nanoparticles were monodispersed and presented growth inhibition activity against *P. aeruginosa* PAO1 and *Bacillus* sp., at 200 μg/ml concentration (Moopantakath et al., 2022).

## 6. Conclusion

Haloarchaea, or extremely halophilic archaea, are a group of microbes with genuine metabolic features. They inhabit and even predominate in various extreme geographical and ecological/environmental conditions, The number of new haloarchaeon discovered through culturable and non-culturable methods and techniques is increasing during the last decades. Metabolites synthesized by several haloarchaeal species are of high interest due to their potential applications in biotechnology Thus, carotenoids are highly efficient as antimicrobials, antioxidants, and food colorants. They have been reported to have diverse biological activities, including anticancer and antimicrobial activities. Among carotenoids, bacterioruberin, carotenoids almost produce by haloarchaea show higher antioxidant activity than most of the referenced carotenoids from plants, yeast or algae. Haloarchaea are also implemented in hypersaline wastewater treatment to degrade hydrocarbons, nitrogen removal, and heavy metal bioremediation and nanoparticle (NP) biosynthesis. On the other hand, enzymes from haloarchaea like amylase, chitinase, lipase, protease, and esterase show high activity at extreme conditions (in terms of pH, temperature etc.) which are of interest for industrial processes. The biosynthesis of nanoparticles by haloarchaea has also described being many of those nanoparticles effective against drug-resistant microbes. The haloarchaea, haloenzymes, and pigments are also widely used in the fermentation of salty foods, cosmetics, food, biomedical sectors, biocompatible bioplastics, and biosensors. Despite such an array of applications, their roles within the cells in natural environments as well as in industrial processes remain unexplored consequently, the research on the metabolites synthesized by these microorganisms must continue through more specialized approaches using high-tech equipments.

## Author contributions

RK and SB designed the study. JM and VA prepared the figures. RK, SB, MI, JM, and VA wrote the manuscript. RK, SB, RM-E, and MD critically revised the manuscript. All authors have read and agreed to the submitted version of the manuscript.
